# Knowledge and preparedness of healthcare providers towards bioterrorism

**DOI:** 10.1186/s12913-021-06442-z

**Published:** 2021-05-05

**Authors:** Abdullah Nofal, Isamme AlFayyad, Nawfal AlJerian, Jalal Alowais, Meshal AlMarshady, Anas Khan, Humariya Heena, Ayah Sulaiman AlSarheed, Amani Abu-Shaheen

**Affiliations:** 1 Emergency Medicine Department, King Saud University Medical City, Riyadh, Saudi Arabia; 2 Research Center, King Fahad Medical City, P.O. Box: 59046, Riyadh, 11525 Saudi Arabia; 3 Medical Referrals Center, Ministry of Health, Riyadh, Saudi Arabia; 4 King Saud Bin Abdulaziz University for health specialities, Riyadh, Saudi Arabia; 5 Emergency and Disaster and Ambulance Services at the Ministry of Health, Riyadh, Saudi Arabia; 6 Adult Emergency Department, King Fahad Medical City, Riyadh, Saudi Arabia

**Keywords:** Bioterrorism, Clinicians, Emergency department, Infectious diseases

## Abstract

**Background:**

Several emergent circumstances require healthcare providers to recognize the unusual and dangerous and pathogenic agents. An in-depth literature review showed that studies about bioterrorism preparedness amongst healthcare providers are lacking. Therefore, this study aimed to investigate the knowledge and preparedness level of first emergency respondents towards bioterrorism events.

**Methods:**

This study has a cross-sectional design and was carried out at the Emergency departments and poison control centers/clinical laboratories three in major tertiary care hospitals in Riyadh, Saudi Arabia. The subjects were randomly selected to complete the self-administered questionnaire to collect study outcomes.

**Results:**

A total of 1030 participants were included in the final data analysis. The mean knowledge score in the basic concepts of bioterrorism and introductory clinical presentations of bioterrorism-related agents was 4.92 ± 1.86 out of 12 points.

Moreover, the findings showed a mean knowledge score of 22.80 ± 3.92 out of 38 in the bioterrorism preparedness and governing policies and procedures. Respondents who received previous training in bioterrorism preparedness had a significantly higher number of perceived benefits than those not sure and without prior training (z = − 2.67, *p* = 0.008) and (z = − 4.4, *p* < 0.0001), respectively. About 79.4% of participants did not have previous training in bioterrorism preparedness, but 68.7% expressed willingness in the institution’s response and control to assist in a bioterrorist attack incident.

**Conclusion:**

Although healthcare professionals have reported their desire to help in bioterrorism events, they need to enhance their knowledge of bioterrorism preparedness.

**Supplementary Information:**

The online version contains supplementary material available at 10.1186/s12913-021-06442-z.

## Background

Bioterrorism is defined as the “intentional use of microorganisms, or toxins derived from living organisms, to produce death or disease in humans, animals or plants” [1, 2]. Bioterrorism might be announced (overt) or unannounced (covert). Announced bioterrorism involves a notification that an agent has been released, leading to the implementation of laws and the involvement of public health agencies from the start. Unannounced bioterrorism might be recognized either once the incubation period has ended or weeks after the occurrence of the event [1].

Depending on the bioterrorism agent and delivery mechanism used, bioterrorism events can target individuals, communities and/or the globe. The international agencies have improved their emphasis on disaster management with four pillars of focus including improving humanitarian values, emergency disaster response, emergency disaster preparedness, and health and community care [3]. Ensuring high-quality reactions to public health emergencies requires evaluating the public health sector’s willingness, readiness, and ability for emergency disasters and bridging and engaging their workforce with governmental efforts to combat bioterrorism events and lessens its impact [4].

The symptoms of clinical presentations of the different illnesses caused by the diverse bioterrorism agents are often very nonspecific and very difficult to differentiate many diseases. In bioterrorism agent attacks, many victims presented simultaneously with similar symptoms, and unusually many of the agents cause a “flu-like” illness [5]. Hospital and clinical laboratories play crucial roles in responding to bioterrorism agent infections and emergent exposures diseases. Hospitals, namely, emergency departments, are the first to receive patients showing specific symptoms, and initially, the illness may present as an unusual disease. Additionally, laboratories will be the first to obtain clinical specimens from patients exposed to bioterrorism agents or infectious diseases. Their rapid identification of these suspected agents and infectious diseases is essential for the prompt recognition and implementation of appropriate responses [1].

Several emergent circumstances require healthcare providers to recognize dangerous and uncommon pathogenic agents [6, 7]. Physicians, nurses, paramedics, and laboratory technicians could be the first healthcare providers exposed to patients with unusual diseases and need to have adequate knowledge to appropriately manage the situation and make decisions to avoid inciting excessive fear and to reduce the exposure of other patients and healthcare providers.

During the 2001 anthrax attack in the United States, clinicians were the first to identify bioterrorism-related anthrax [8–10]. Early recognition is the most vital stage for an efficient surveillance system. The Director of the Center for Disease Control and Prevention (CDC) reported the following:

“*For this frontline surveillance system to function at its best, all clinicians, regardless of their specialty, must have enough basic information about the clinical manifestations of infections caused by the select agents of bioterrorism to raise their suspicion when they see a patient with a compatible illness*” [11].

The ability to respond efficiently to any bioterrorism attack and its consequences has become a challenge that necessitates a massive national effort. Decreases in morbidity and mortality can be accomplished by prompt medical responses, which need planning, preparation, and continuous training [1]. After the release of anthrax in the United States in 2001, the European Union prepared a program on preparedness and response to chemical and biological agent attacks to increase the collaboration between its member states for the assessment of risks, alarming, intervention, storing of necessary means, and foster research in the field of bioterrorism [12]. Since then, several national activities in Europe were also implemented [12–14].

In Saudi Arabia, disaster management plans began 80 years ago. They had been constantly improving ever since to manage natural disasters that have historically taken place, particularly during Hajj (the most significant religious mass gathering in the world). The national emergency management plan is arranged and managed by the General Directorate of Civil Defence (GDCD). The scope of the GDCD includes organizing the national alert system in the case of disasters, controlling key infrastructure, Protection of victims and giving essential life-support measures in affected areas, controlling hazardous areas, arrange between organizations that have different and various resources and specialties to improve disaster response [15].

The Association of American Medical Colleges (AAMC) in 2001 assigned an expert panel to set learning objectives on bioterrorism for medical students [16]. The panel recommended integrating the learning objectives related to bioterrorism events in the basic science and medical curricula. The objectives about the primary science curriculum included natural bioterrorism events, core concepts of microbiology, pathology, biochemistry, physiology, pathophysiology, pharmacology. The medical school must ensure sufficient knowledge of bioterrorism agents, routes of exposure, medical management in combating injuries and illnesses, prevention strategies, and specific Category A and B biologic agents and general classes of chemicals [16]. However, there is a lack of emergency disasters in the undergraduate medicine programs in Saudi Arabia due to the scarcity of specialized professionals as a significant obstacle [17].

Furthermore, the American Association of Colleges of Nursing and the International Nursing Coalition for Mass Casualty Education established a set of bioterrorism preparedness competencies for nursing [18]. To support the bioterrorism preparedness curriculum for healthcare providers, the Health Resources and Services Administration sponsored some universities and educational organizations as part of its core bioterrorism curriculum program [19]. Despite all of these efforts, low levels of perceived preparedness have been documented in the literature [20–22]. A study conducted by Chen et al. (2001) using a mailed survey among a random sample of members physicians of the American Academy of Family Physicians showed that 24% agreed that they could identify a bioterrorism event, and 19% reported that they could respond efficiently to a bioterrorism event [20]. However, Chen‘s study survey was mailed before reporting anthrax outbreaks to the Centers for Disease Control and Prevention and before developing the curriculum by the AAMC. Another study conducted on healthcare professionals revealed that only 23% of respondents felt “confident” to provide the needed care during a bioterrorism event [21]. Similarly, a national survey demonstrated that only 21% of physicians felt ready to deal with a bioterrorism event [22].

An in-depth literature review, using MEDLINE/PubMed, CINAHL, google scholar, and Embase for relevant published studies from Saudi Arabia, showed that studies about bioterrorism preparedness amongst healthcare providers from the Middle East, including Saudi Arabia, are lacking. Therefore, we carried out this study to investigate the knowledge and preparedness level of emergency first responders for bioterrorism events, including emergency department (ED) healthcare providers and poison control center/clinical laboratory staff.

## Methods

A cross-sectional study was conducted at three EDs and clinical laboratories in three major tertiary care hospitals in Riyadh, Saudi Arabia. All poison control center/clinical laboratory staff involved in dealing with bioterrorism events and ED healthcare providers, including physicians, nurses, paramedic/emergency medical services (EMD) team with at least one year of clinical experience, were eligible for recruitment into the study. Subjects with less than one year of clinical experience were excluded from this study.

Data were collected using a structured questionnaire based on a review of the literature of related studies [23, 24]. A pilot study was conducted on 30 participants to validate the clarity and readability of the study tool. The subjects were randomly invited to participate in this study by the study coordinator. The participants were asked to put the completed questionnaires in an anonymous box kept at a designated place in their respective departments to protect their identity and to ensure the anonymity and confidentiality of the data.

### Patient and public involvement

No patient involved.

### Data tool description

The survey instrument consisted of four subscales measuring the following: (i) bioterrorism preparedness knowledge, (ii) perceived benefits, (iii) perceived barriers, and (iv) bioterrorism preparedness education receipt and responses.

The bioterrorism knowledge component of the questionnaire consisted of 38 true or false items, with one point awarded for each correct answer; higher scores indicated higher knowledge levels. The maximum possible score for bioterrorism knowledge was 38.

Additionally, 12 multiple choice knowledge-based questions with a single correct answer that was worth 1 point were added from a study conducted by Katz et al. The questions concentrated on bioterrorism agents, syndromic surveillance, and the recognition of disease manifestations of CDC “Category A” agents to evaluate basic concepts in bioterrorism and clinical presentations of bioterrorism agents, with additional questions specifically related to the identification of smallpox and anthrax and control measures for smallpox [23].

Survey questions regarding the perceived benefits of bioterrorism preparedness education and barriers were based on a five-point Likert-type scale (strongly agree, agree, neutral, disagree, and strongly disagree). Scores of the benefit and barrier items were summed to provide overall scores for the benefits and barriers subscales. Higher scores on these subscales indicated a higher perceived benefit of receiving training (maximum score: 30) or a higher number of perceived barriers to education participation (maximum score: 70) [24, 25].

The instrument assessed bioterrorism education and response by asking the study participants about the following: (i) prior bioterrorism preparedness training and the areas that they need training in; and (ii) if the respondent would be willing to assist the institution in its bioterrorism response and control efforts.

The Cronbach’s alpha was 0.86 for the perceived benefits, 0.91 for the perceived barriers, and 0.88 for bioterrorism knowledge, providing internal consistency for these subscales [24, 25].

Additionally, the questionnaire included demographic questions, such as profession (ED physician, infectious disease physician, ED nurse, and paramedic), age, gender, highest education level, workplace, and whether they were a member of an employer disaster planning committee.

### Ethical considerations

The study was reviewed and approved by the institutional review boards of the three hospitals where the research was conducted:

1. King Fahad Medical City Institutional Review Board, King Fahad Medical City hospital, Riyadh, Saudi Arabia.
2. King Saud University Medical City Institutional Review Board, King Saud University Medical City hospital, Riyadh, Saudi Arabia.
3. Ministry of National Guard Institutional Review Board, Ministry of National Guard hospital, Riyadh, Saudi Arabia.

Also, written informed consent was obtained from each participant.

### Sample size calculation/justification

Only 20% of physicians or nurses had previous training in bioterrorism preparedness. Assuming that 20% of the subjects in a population have the factor of interest, the study would require an estimated sample size of *n* = 246, with 5% absolute precision and 95% confidence.

After applying a finite population correction (FPC), the estimated sample size for the respective study settings of ED healthcare providers was *Hospital 1* = 142, *Hospital 2* = 144, *Hospital 3* = 156. Furthermore, the response rate in the reference study [16] was 45% for physicians and 53% for nurses, with an average response rate of 50%, which was adjusted for the proposed study. These considerations increased the sample size to twice the FPC sample size; after that, the sample size for the four clusters was *Hospital 1* = 284 *Hospital 2* = 288 and *Hospital 3* = 312, further apportioned into four strata: physicians, nurses and paramedics/EMS. We included all poison control centres/clinical laboratory staff dealing with bioterrorism events, as there were only a few individuals.

### Statistical analysis

SPSS version 24.0 was used in analyzing the data. Continuous variables (age and experience) were placed into groups, as shown in Table 1. Descriptive statistics are presented as percentages, frequencies, averages, and standard deviations, as appropriate. A comparison of the distribution of categorical outcomes was performed by applying chi-square tests. The Kolmogorov-Smirnov test was used to overall knowledge-based test scores and variables for perceived benefits and barriers to receiving bioterrorism preparedness education. The test indicated that the distribution of all of these variables deviated from the normal distribution. Therefore, nonparametric tests were used to test whether there is a significant difference in the average scores of these variables based on respondent demographic characteristics. Accordingly, the Kruskal–Wallis test is usually applied to compare independent mean outcomes of categorical variables that contained more than two categories. The Mann–Whitney test is used to compare independent mean outcomes by dichotomous variables that included two types. Statistical significance was considered at a *P*-value of less than0.05.

**Table 1:** Descriptive statistics of respondents’ demographic characteristics

Variables	n (%)
**Sex**	
▪ Male	476 (46.4)
▪ Female	550 (53.6)
**Hospital**	
▪ Hospital 1	410 (39.8)
▪ Hospital 2	293 (28.5)
▪ Hospital 3	327 (31.7)
**Department/unit**	
▪ Adult Emergency	538 (52.2)
▪ Paediatric Emergency	322 (31.3)
▪ Paramedic/EMS	129 (12.5)
▪ Poison Control Centre/Clinical Laboratory Department	40 (3.8)
**Profession**	
▪ Physician	351 (34.1)
▪ Nurse	516 (50.1)
▪ Paramedic/EMS	131 (12.7)
▪ Poison Control Centre/Clinical Laboratory Department	32 (3.1)
**Age (years)**	
▪ ≤ 30	429 (43.2)
▪ 31 to 40	365 (36.8)
▪ 41 to 50	154 (15.5)
▪ 51 to 60	40 (4.0)
▪ ≥ 61	4 (0.4)
**Highest level of education**	
▪ Diploma	126 (12.3)
▪ Bachelor’s	672 (65.6)
▪ Master’s degree	62 (6.0)
▪ Doctorate	58 (5.7)
▪ Subspecialty or Fellowship	107 (10.4)
**Experience at the current institution (years)**	
▪ ≤ 10	872 (86.6)
▪ 11 to 20	121 (12.0)
▪ ≥21	14 (1.4)
**Total years of experience**	
▪ ≤ 10	684 (68.5)
▪ 11 to 20	244 (24.4)
▪ ≥21	70 (7.0)

Because of missing data in responses, items have various denominators

## Results

The study involved a total of 1030 participants. Four hundred twenty-two (43.2%) respondents were less than or equal to 30 years old, had a bachelor or higher educational levels (*n* = 899, 87.7%), and had less than or equal to 10 years of experience at the current institution (*n* = 872, 86.6%) (Table 1).

Approximately 83% of respondents were not currently or had never been a disaster planning committee member. About 46% of respondents were not aware of the hospital policy and procedure during a bioterrorism attack (*n* = 462, 45.7%).

### Bioterrorism knowledge-based multiple-choice questions

Respondents had a mean correct score of 4.92 ± 1.86 (*standard deviation*) out of 12 questions (40%) (*median* = 5). Respondent scores ranged from 0 to 11 points. Only 35.3% of respondents scored above the median total score.

The question with the least correct responses by study respondents was “identification of diseases for which persistent environmental spores are a concern after a bioterrorism event” (21.9%), followed by “policies to notify the Ministry of Health when a physician saw a patient he or she suspects of having anthrax or smallpox” (26.2%), “recognition of clinical features distinguishing anthrax from an upper respiratory tract infection” (28.7%) and “identifying all clinical features of smallpox in which it may spread out without direct or indirect contact with open lesions” (30.5%). While, the top three questions with the most correct responses were “the identification of the deadliest form of anthrax, which is inhalation anthrax,” followed by “What is a critical measure in preventing contact transmission of vaccinia virus (the agent used in the currently licensed smallpox vaccine)?” and “Which of the following diseases has the potential for the person-to-person spread?” (Table 2).

**Table 2:** Bioterrorism knowledge-based multiple-choice questions

Questions	n (%)
**Q1. The deadliest form of anthrax is:**	
▪ **Inhalation**	**783 (76.8)**
▪ Cutaneous	111 (10.9)
▪ Gastrointestinal	72 (7.1)
▪ Bubonic (swollen lymph nodes)	53 (5.2)
**Q2. What is a critical measure in preventing contact transmission of vaccinia virus (the agent used in the currently licensed smallpox vaccine)?**	
▪ **Thorough hand washing after contact with the vaccination site**	**534 (53.3)**
▪ Isolation of the vaccinated person	200 (20.0)
▪ Use of a porous bandage to cover the vaccination site	99 (9.9)
▪ Application of the vaccine at an anatomic site normally covered by clothing	94 (9.4)
▪ Antibacterial ointment applied to the vaccination site	74 (7.4)
**Q3. “Which of the following diseases has the potential for the person-to-person spread?”**	
▪ **Smallpox and plague**	**526 (51.5)**
▪ Anthrax and plague	414 (40.5)
▪ Plague and botulism	41 (4.0)
▪ Botulism and brucellosis	41 (4.0)
**Q4. Which of the following features help to distinguish the rash of smallpox from that of chickenpox?**	
▪ **The smallpox rash is centrifugal (the majority of lesions are on the face and extremities), while the rash in chickenpox is central (the majority of lesions lie on the trunk).**	**471 (47.2)**
▪ The initial smallpox lesions coincide with the onset of fever, while the fever in chickenpox precedes the rash by 2–3 days	260 (26.1)
▪ Various stages of lesion progression can be found at any one single location on a smallpox patient, while the lesions of chickenpox tend to all occur at the same stage of development.	191 (19.1)
▪ Lesions rarely occur on the palms and soles in smallpox, while lesions commonly occur on the palms and soles in chickenpox.	76 (7.6)
**Q5. The most common early presenting syndrome associated with the majority of high-risk (“Category A”) bioterrorism-associated diseases (i.e., anthrax, botulism, plague, smallpox, tularaemia, and viral haemorrhagic fevers) is:**	
▪ **Influenza-like illness**	**415 (40.8)**
▪ Fever and rash	380 (37.4)
▪ Acute bloody diarrhoea	179 (17.6)
▪ Acute hepatitis	42 (4.1)
**Q6. A pathognomonic chest X-ray finding of advanced inhalation anthrax is:**	
▪ **Widened mediastinum**	**429 (42.6)**
▪ Cavitation	293 (29.1)
▪ Normal chest X-ray despite dyspnoea and tachypnea	286 (28.4)
**Q7. Epidemiologic features of a plague outbreak that may indicate an intentional release of the plague organism include:**	
▪ **Location of infections outside of areas of known enzootic infection**	**391 (39.5)**
▪ Occurrence in persons with known health risks such as chronic pulmonary disease	336 (33.9)
▪ Occurrence in areas with prior reported rodent deaths	263 (26.6)
**Q8. Which of the following are high biological terrorism threats because of substantial morbidity and mortality, ease of production, efficient dissemination, stability in aerosol, or high infectivity?**	
▪ **Anthrax, smallpox, botulism, and plague**	**331 (32.6)**
▪ Anthrax, chickenpox, botulism, and plague	291 (28.6)
▪ Anthrax, smallpox, chickenpox, and plague	250 (24.6)
▪ Anthrax, smallpox, mumps, and plague	144 (14.2)
**Q9. Smallpox has all of the following clinical features EXCEPT:**	
▪ **The virus can only be spread through direct or indirect contact with open lesions (e.g., by touching an infected lesion or by contact with infected clothing or bedding).**	**307 (30.5)**
▪ During the incubation period, the infected person looks and feels healthy and cannot infect others	279 (27.7)
▪ Infectivity is highest after the fever has begun and during the first 7–10 days following the appearance of the rash.	287 (28.5)
▪ The incubation period ranges from 7 to 17 days.	135 (13.4)
**Q10. Which of the following symptoms is/are not commonly found in inhalation anthrax, and if present, could help to differentiate an upper respiratory tract infection from anthrax?**	
▪ **Rhinorrhoea and sore throat**	**293 (28.7)**
▪ Meningeal signs	389 (38.1)
▪ Dyspnoea	191 (18.7)
▪ Vomiting	140 (14.5)
**Q11. According to KFMC policies, a physician who sees a patient he or she suspects of having anthrax or smallpox must notify the Ministry of Health:**	
▪ **By phone as soon as the provisional diagnosis is established**	**265 (26.2)**
▪ By phone as soon as the suspected diagnosis has been laboratory confirmed	390 (38.5)
▪ By mail, phone, or fax within 72 h	184 (18.2)
▪ Immediately after receiving written permission from the patient (or his/her legal guardian)	174 (17.2)
**Q12. According to KFMC policies, a physician who sees a patient he or she suspects of having anthrax or smallpox must notify the Ministry of Health:**	
▪ **By phone as soon as the provisional diagnosis is established**	**265 (26.2)**
▪ By phone as soon as the suspected diagnosis has been laboratory confirmed	390 (38.5)
▪ By mail, phone, or fax within 72 h	184 (18.2)
▪ Immediately after receiving written permission from the patient (or his/her legal guardian)	174 (17.2)

Because of missing data in responses, items have various denominators

The questions have been ordered from the most to the least correct answers

Moreover, statistically significant differences were detected in overall test results by department (*χ*^2^ = 16.98, *p* = 0.002). In particular, respondents in the adult emergency and paediatric departments did better on knowledge-based multiple-choice questions (MCQ) than those working in the EMS/paramedic department (*p* = 0.016; 0.003, respectively) and poison control centre/clinical laboratory department (*p* = 0.014; 0.006, respectively). Furthermore, there were statistically significant differences in overall test scores among respondents based on profession (*χ*^2^ = 18.36, *p* < 0.001). Physicians and nurses did better on the general knowledge-based test than paramedic/EMS and poison control center/clinical laboratory department staff (*p* = 0.001, 0.035, 0.002, and 0.009, respectively). Overall test scores also significantly differed among respondents based on educational level (*χ*^2^ = 10.13, *p* = 0.038). Respondents who held doctorate degrees scored higher than those who held diplomas (*p* = 0.006) and bachelor’s (*p* = 0.004) degrees. Additionally, respondents who received prior training concerning preparedness of bioterrorism had significantly higher scores than those who had not (*χ*^2^ = 13.37, *p* = 0.001).

### Bioterrorism preparedness knowledge true or false questions

Respondents had a mean correct score of 22.80 ± 3.92 (*standard deviation*) out of 38 questions (60%) (*median* = 23) on bioterrorism knowledge. Respondent scores ranged from 11 to 33 points. Approximately 61% of the respondents scored above the median total score. The question with the least correct responses by the study respondents was “chain of custody documentation is required for tracking patient specimens following a bioterrorism attack” (6.2%). This was followed by “airborne-spread diseases require the use of a negative pressure room in all settings” (10.3%), “quarantine will be instituted after a bioterrorism attack involving any contagious disease” (11.0%), and “run-off water from patient decontamination following a bioterrorism attack must be contained” (11.0%). However, most respondents correctly answered the question of “recent travel history, occupation, and vaccination history of victims that will be needed as a part of the epidemiological investigation of a bioterrorism attack” (92.3%) (Table 3).

**Table 3:** Bioterrorism Preparedness Knowledge true or false questions

No.	Questions	n (%)	Correct answer
1	A recent travel history, occupation, and vaccination history of victims will be needed as part of the epidemiological investigation of a bioterrorism attack.	938 (92.3)	True
2	The four phases of emergency management include mitigation, preparedness, response, and recovery.	936 (91.8)	True
3	Biological agents can be dispersed via food, water, direct contact, or through aerosolization.	935 (91.8)	True
4	If you have children, back-up childcare should be arranged as part of your bioterrorism response plan.	914 (90.2)	True
5	Immunocompromised individuals will be more at risk for disease following a bioterrorism attack than young, healthy adults.	913 (90.0)	True
6	Patient isolation should be based on the route of disease transmission.	901 (88.1)	True
7	Many of the potential bioterrorism agents cause upper respiratory symptoms.	898 (88.6)	True
8	Both acute and long-term mental health effects, such as anxiety and post-traumatic stress disorder, can be expected to rise after a bioterrorism attack.	897 (88.1)	True
9	Personal protective equipment should be chosen based on the task being performed and the patient’s isolation precautions category.	882 (86.1)	True
10	All nurses (except those working in public health) should report suspected bioterrorism attacks to the local health department.	881 (86.6)	True
11	Weather conditions can affect the length of time that aerosolized biological particles remain airborne.	879 (86.3)	True
12	Young children and the elderly are two of the most vulnerable populations to the effects of a bioterrorism attack.	873 (85.6)	True
13	Environmental decontamination procedures depend upon the agent released.	865 (85.5)	True
14	Plans for back-up transportation should be arranged as part of nurses’ response plans.	860 (84.6)	True
15	Patient specimens should be hand-carried to the laboratory during the response to a bioterrorism attack; automated tube systems should not be used.	848 (83.8)	True
16	A sudden influx of patients with flu-like symptoms may be the earliest indication of a bioterrorism attack.	838 (82.2)	True
17	A large number of patients presenting with a rapidly fatal disease may indicate a bioterrorism attack has occurred.	835 (82.6)	True
18	Nurses do not need a personal response plan for bioterrorism because their facility will have a disaster plan.	648 (64.9)	False
19	If you have been vaccinated against the disease that the patient has, you do not need to wear personal protective equipment when providing nursing care to them.	645 (63.9)	False
20	Only bleach should be used to disinfect environmental sources indoors following a bioterrorism attack.	634 (62.3)	False
21	Nurses’ routine job duties will not be impacted by a bioterrorism attack.	632 (62.3)	False
22	Vaccination administration following a bioterrorism attack will be similar to day-to-day immunizations.	536 (53.0)	False
23	All patients infected with a disease will have symptoms.	518 (51.0)	False
24	Bioterrorism attacks must not be reported until they are confirmed.	512 (50.2)	False
25	Procedures for biological and chemical patient decontamination are the same.	507 (49.7)	True
26	Guidelines about removing patients from isolation are the same after a bioterrorism attack as routine procedures.	483 (47.4)	False
27	It is unsafe to cohort patients (putting patients with the same disease in the same room) during the response to a bioterrorism attack.	480 (47.4)	False
28	Only police, emergency medical services, and fire protection professionals will use the incident command system to communicate during a bioterrorism attack.	400 (39.3)	False
29	The use of alcohol-based products is an effective means of removing debris from the hands of victims exposed to a biological agent.	349 (34.5)	False
30	Duct-taping your windows will prevent the infiltration of infectious particles into your house following an aerosol release.	329 (32.3)	False
31	Patient decontamination for bioterrorism includes the use of bleach as a disinfectant.	323 (31.9)	False
32	The response actions for emerging infections, such as SARS and monkeypox, are very different from those for bioterrorism.	290 (28.5)	False
33	Nurses caring for patients with diseases spread by respiratory droplets must wear N-95 masks.	211 (20.8)	False
34	Prompt initiation of post-exposure prophylaxis will prevent all patients from developing the disease.	195 (19.3)	False
35	A quarantine will be instituted after a bioterrorism attack involving any contagious disease.	112 (11.0)	False
36	Run-off water from patient decontamination following a bioterrorism attack must be contained.	111 (11.0)	False
37	Airborne-spread diseases require the use of a negative pressure room in all settings.	104 (10.3)	False
38	Chain of custody documentation is required for tracking patient specimens following a bioterrorism attack.	61 (6.2)	False

Because of missing data in responses, items have various denominators

The questions have been ordered from the most to the least correct answers

Moreover, the results showed that differences in general test results based on the department were statistically significant (*χ*^2^ = 30.16, *p* < 0.0001). In particular, respondents in the adult emergency department received a higher mean score on knowledge-based true or false questions than those who worked in the pediatric emergency (*p* < 0.001) and EMS/paramedic (*p* = 0.001) departments. Furthermore, respondents showed strong, statistically significant differences in total test scores based on profession (*χ*^2^ = 16.42, *p* = 0.001). Physicians had significantly better overall results than nurses and paramedic/EMS (*p* < 0.0001, 0.003, respectively). Also, there were significant differences in overall test scores among respondents in different age categories (*χ*^2^ = 13.29, *p* = 0.010). That is, respondents had successively better results in each age group from 31 to 40 years through 61 years or above than those in the 31 years and less group (*p* = 0.018, 0.029, 0.010, and 0.028, respectively). Moreover, respondents over 61+ years scored significantly better than those aged 31–40 (*p* = 0.046). Differences in overall scores by education levels were not significant (*χ*^2^ = 15.22, *p* = 0.004). Respondents with higher education levels had better overall test scores. That is, respondents with master’s, doctorate, and subspecialty or fellowship degrees scored better than diploma holders (*p* = 0.002, 0.004, 0.007, respectively). Respondents with a master’s degree also did better on the real test than those with a Bachelor’s degree (*p* = 0.027). Respondents with fewer years of experience (≤ 10 years) at the current institution as well as total years of experience had better results than others (11–20 and 21+ year category), and these differences were statistically significant (*p* = 0.005, 0.003). Finally, higher and statistically significant scores achieved by respondents who received previous training regarding bioterrorism preparedness than those who had not (*χ*^2^ = 7.99, *p* = 0.018).

### Perceived benefits and barriers to bioterrorism education

Most respondents (96.2%, *n* = 969) stated that they agreed with at least one benefit to becoming better prepared for bioterrorism, and approximately 53% (*n* = 530) agreed with all six items on perceived benefits. The highest level of agreement was attached to “the benefits of getting better prepared for bioterrorism will decrease my chances of getting sick/dying after a bioterrorism attack” and “will decrease my patients’ risk of getting sick/dying after a bioterrorism attack” (4.2 (81.4%) and 4.8 (80.9%), respectively). Respondents’ agreement with each of the perceived benefit statements is shown in Table 4.

**Table 4:** Perceived Benefits and Barriers to Bioterrorism Education

No	Statement	Mean (SD)	Frequency of agreement with the statement, n (%)
1	Getting better prepared for bioterrorism will decrease my chances of getting sick/dying after a bioterrorism attack.	4.20 (1.01)	831 (81.4)
2	Getting better prepared for bioterrorism will decrease my patients’ risk of getting sick/dying after a bioterrorism attack.	4.18 (0.97)	825 (80.9)
3	Getting better prepared for bioterrorism makes me feel safer.	4.13 (0.94)	788 (77.7)
4	Getting better prepared for bioterrorism will decrease my family’s risk of getting sick/dying after a bioterrorism attack.	4.12 (0.97)	819 (80.4)
5	Bioterrorism preparedness advances my knowledge	4.07 (1.00)	787 (77.5)
6	Getting better prepared for bioterrorism will increase my chances of detecting an attack before surveillance would recognize it	4.05 (0.98)	769 (75.4)
7	I do not know where to get bioterrorism preparedness training.	3.53 (1.07)	565 (55.5)
8	There are no bioterrorism-related disaster exercises available.	3.33 (1.07)	413 (43.8)
9	There are no training opportunities available on bioterrorism preparedness.	3.29 (1.12)	439 (43.1)
10	There is no administrative or financial support for bioterrorism preparedness training for me at my job	3.28 (1.04)	399 (39.4)
11	Bioterrorism training is too expensive	3.09 (0.87)	228 (22.6)
12	Bioterrorism training will take too long	3.03 (0.91)	235 (23.4)
13	I have no interest in bioterrorism preparedness.	2.38 (1.26)	207 (20.5)
14	I feel uncomfortable/stressed when thinking about bioterrorism.	2.98 (1.09)	333 (32.9)
15	My work schedule does not provide time for bioterrorism training.	2.95 (1.12)	295 (29.2)
16	There is little one can do to lessen the impact of a bioterrorism attack.	2.79 (1.13)	255 (25.2)
17	I am too busy for bioterrorism training.	2.79 (1.13)	245 (24.2)
18	Bioterrorism preparedness is not within the scope of my responsibilities.	2.54 (1.12)	189 (18.7)
19	Bioterrorism training is all the same; I am not learning anything new	2.69 (1.02)	170 (16.8)
20	Bioterrorism preparedness is not currently a priority for me.	2.59 (1.19)	221 (21.8)

Because of missing data in responses, items have various denominators

The questions have been ordered from the most to the least correct answers

Benefits mean score was 24.85 (range = 6 to 30, standard deviation = 5.03) out of a total of 30 points, which indicates that the majority of respondents expressed their agreement with almost all of the benefits of obtaining education concerning bioterrorism preparedness.

There were no statistically significant differences between the stated number of perceived benefits and department, profession, age, years of experience at the current institution, total years of experience, willingness to assist in the case of a bioterrorism attack, and being a member of a disaster planning committee. However, male respondents stated a significantly higher number of perceived benefits than their female counterparts (*z* = − 2.20, *p* = 0.013). There were also significant differences in perceived benefits based on education levels (*χ*^2^ = 13.12, *p* < 0.011). Respondents with master’s and doctorate degrees reported a higher number of perceived benefits than respondents with a diploma degree (*p* = 0.004, 0.014, respectively). Respondents with a doctorate also reported a higher number of perceived benefits than those with a bachelor’s degree (*p* = 0.017). Moreover, respondents showed significant differences in perceived benefits according to prior training (*χ*^2^ = 19.54, *p* < 0.0001). That is, respondents who received previous training in bioterrorism preparedness reported a significantly higher number of perceived benefits than those who were not sure and who did not have prior training (*z* = − 2.67, *p* = 0.008 and *z* = − 4.4, *p* < 0.0001, respectively). Additionally, respondents showed significant differences in perceived benefits according to the awareness of hospital policies and procedures during a bioterrorism attack (*χ*^2^ = 8.68, *p* = 0.013). That is, respondents who were already aware of hospital policies and procedures during a bioterrorism attack reported a significantly higher number of perceived benefits than those who did not know whether they were aware or not (*z* = − 2.43, *p* = 0.015).

Respondent agreements with each of the perceived barrier statements are shown in Table 4. Respondents reported various perceived barriers, and we mentioned the most frequent ones. First, I do not know where to get bioterrorism preparedness training. Second, there are no bioterrorism-related disaster exercises available. Third, there are no training opportunities available on bioterrorism preparedness. Finally, there is no administrative, financial support for bioterrorism preparedness training for me at my job.

No significant differences in the number of perceived barriers and respondents’ department, profession, age, gender, level of education, years of experience at the current institution, total years of experience, and willingness to assist in the case of a bioterrorism attack were detected.

Respondents who were members of a disaster planning committee reported significantly fewer perceived barriers than their non-member counterparts (*z* = − 2.69, *p* = 0.007). Additionally, respondents showed significant differences in perceived barriers according to their awareness of hospital policies and procedures during a bioterrorism attack (*χ*^2^ = 20.61, *p* < 0.001). Respondents who were already aware of hospital policies and procedures during a bioterrorism attack reported a significantly higher number of perceived barriers than those who were not aware of such policies (*z* = − 4.34, *p* < 0.001) and did know whether they were aware or not (*z* = − 2.78, *p* = 0.005). Furthermore, there were statistically significant differences in perceived barriers according to previous training (*χ*^2^ = 42.54, *p* < 0.0001). That is, respondents who already had previous training in bioterrorism preparedness indicated a significantly lower number of perceived barriers than those who were not sure or had no prior training (*z* = − 6.49, *p* < 0.0001 and *z* = − 4.32, *p* < 0.0001, respectively).

### Respondent training

Approximately 79.4% of participants had not trained previously concerning bioterrorism preparedness (*n* = 789, 79.4%). Typically, a small proportion of them had not received previous training and education in such situations (*n* = 89, 8.8%). Approximately 11.9% of them were not sure whether they had already received training and education or not (*n* = 118). More than two-thirds of respondents expressed their willingness to assist in the institution’s response to help control a bioterrorist attack (*n* = 683, 68.7%). However, 13.5 and 17.8% of them were not willing or not sure how to provide such assistance, respectively.

### Prospective to obtain bioterrorism training in the future

Most respondents expressed their interest in receiving training in the eleven areas they needed training listed in the questionnaire. The four most commonly required areas for training were as follows: (1) “recognition of an illness or injury in humans as potentially resulting from exposure to a bioterrorist agent” (*n* = 818, 83.9%); (2) “institutional laws and statutes relating to public health measures” (*n* = 771, 81.5%); (3) “safety measures to be taken by a public health responder in a bioterrorism event, including the use of protective equipment” (*n* = 794, 81.4%); and (4) “basic education regarding biological incidents” (*n* = 767, 80.3%). Table 5 shows respondent needs for each training area.

**Table 5:** Responses to Receiving Bioterrorism Education in the Future

No.	Statement	n (%)
1.	Recognition of an illness or injury in humans as potentially resulting from exposure to a bioterrorism agent.	818 (83.9)
2.	Safety measures to be taken by a public health responder in a bioterrorism event, including the use of protective equipment.	794 (81.4)
3.	KFMC laws and statutes relating to public health measures.	771 (81.5)
4.	Basic education regarding biological incidents.	767 (80.3)
5.	How the public health system works in Saudi Arabia.	757 (78.1)
6.	Isolation and decontamination procedures	749 (79.9)
7.	Disease investigation and reporting/epidemiologic methods	748 (77.9)
8.	Surveillance (including syndromic surveillance) for a bioterrorism agent.	748 (79.2)
9.	Who to call if a bioterrorism event is suspected.	728 (77.9)
10.	How to access clinical information about bioterrorism.	716 (76.7)
11.	Laboratory diagnosis of a bioterrorism agent.	696 (73.8)

Because of missing data in responses, items have various denominators

The questions have been ordered from the most to the least correct answers

## Discussion

The results from the present study revealed that respondents had poor bioterrorism knowledge related to detecting diseases associated with bioterrorism and their symptoms, as well as institutional policies for notifying the Ministry of Health, which is reflected in their low average MCQ test scores. Most respondents were nurses working in adult emergency departments who held a bachelor’s degree. Other studies have reported weak levels of bioterrorism knowledge. A study by Katz et al. [23], for instance, showed that respondents scored approximately 8.4 (SD = 1.8) out of 12 points. However, this average was higher than that of the respondents in the current study, meaning that the other study reported lower weakness levels of bioterrorism knowledge based on MCQs.

Moreover, the observed differences in this test performance were attributed to differences in respondents’ workplace, department, profession, educational levels, and prior bioterrorism preparedness training. These results were similar to Katz et al. [23], who showed that respondents’ performance significantly differed by profession, work experience, education levels, and prior training. Also, they showed that the results also varied by age group, which contradicts our findings.

Another supporting evidence for weak bioterrorism knowledge was found by the poorer average scores of the true or false question tests. Various studies reported poor performance on the true or false question tests. For example, a study by Terri and Lisa [25] indicated that respondents achieved low average test scores (mean = 27.7, SD = 4.37; out of 38 points). However, respondents in our study scored lower than the participants in the survey by Terri and Lisa [25]. Moreover, our study findings were similar to those of De Felice et al. [17], Katz et al. [23], and Rose and Larrimore [22]. Responses to the overall test questions varied significantly by workplace, department, profession, age group, education level, experience, and prior bioterrorism preparedness training. The findings from the present study were confirmed by the results of Terri and Lisa [25]. In general, the findings confirmed those of previous studies, which indicated that the knowledge of bioterrorism preparedness across different professions, departments, locations, education levels, and work settings is generally low and poor [23–28].

Almost all respondents who participated in this study agreed that bioterrorism preparedness education would help them to be better prepared for bioterrorism for various reasons. These include but are not limited to “getting better prepared for bioterrorism will decrease their chances of getting sick/dying after a bioterrorism attack” and “will decrease my patients’ risk of getting sick/dying after a bioterrorism attack” were among the top-cited items. Even though there was a high level of agreement on the stated perceived benefits to obtaining education or training, most respondents in our study pointed out that they did not receive any previous bioterrorism preparedness education or training programs. These results were similar to those of an investigation by Katz et al. [23], which showed that 21.4% of physicians and 20.4% of nurses received training. Moreover, a study by Terri and Lisa [25] showed that 8.4% of participants indicated that they had already participated in the free nursing bioterrorism preparedness training CD-ROM program. Meanwhile, the observed differences in respondents’ perceived benefits were associated with gender, education levels, prior training, and awareness about hospital policies during a bioterrorism attack. Differences by gender and disaster planning committee membership were evident, but differences based on education levels, age, and prior training were not apparent in the study by Terri and Lisa [25].

Most respondents in the present study recognized several barriers to getting bioterrorism education. Respondents generally did not know where to enroll in training programs concerning bioterrorism preparedness, and the absence of available bioterrorism-related disaster exercises was among the most frequently reported barriers. Furthermore, the lack of training opportunities and the administrative and financial support for bioterrorism preparedness training at their workplace were also among other most cited barriers. The study by Terri and Lisa [25] indicated similar barriers but with different frequencies and showed that barriers varied significantly by work setting or specialty. Based on these barriers, healthcare professionals must be better prepared for emergencies. Therefore, healthcare service providers must increase professionals’ access to bioterrorism preparedness education by identifying training locations, providing bioterrorism-related disaster exercises, and providing the necessary financial support to encourage employees to participate bioterrorism-related training programs. Suitable improvements and interventions should be developed for specific barriers to education indicated by respondents in the present study. Accordingly, healthcare professionals must be provided with supplementary information concerning available training, bioterrorism preparedness programs and learning resources relevant to all types of disasters. Healthcare professionals could be supported in further improving their education by providing financial support such as fellowships or research funds.

Even though most respondents in the current study had never attended prior training in bioterrorism preparedness, they expressed their willingness to assist in the institution’s response to help to control a bioterrorism attack. However, being willing to assist, not being willing to assist, or not being sure about being ready to assist in the case of bioterrorism attacks had no effects on knowledge-based tests, perceived benefits, and barriers, which confirmed the previous findings reported by Katz et al. [25].

The majority of respondents expressed their intention to receive future education or training to become better prepared in case of a bioterrorism attack and identified various areas of needed education. Recognizing an injury or illness of humans that possibly resulted from exposure to a bioterrorism agent and institutional-related acts and laws to the measures of public health were among the most cited areas for future education. Another commonly cited area for future education was safety measures to be performed by a public health responder in a bioterrorism incident, including the use of protective equipment and primary education regarding biological incidents. Previous studies indicated that most participants planned to attend bioterrorism education and training programs in the future [25]. This highlights the need to develop various bioterrorism educational programs for workers and health care service providers. Such education must be based on competency and linked to goals that can be utilized to examine healthcare workers’ performance [29]. Accordingly, education programs related to bioterrorism preparedness should be accessible in a diversity of modalities, such as lectures and online courses, to meet the workers and health service providers [25]. It may be integrated into undergraduate courses in university-related programs [23]. An earlier study by Fatima indicated that most nursing students in Saudi Arabia were motivated to know disaster plans (69.8%), and they had used the internet to find information regarding bioterrorism or emergency preparedness (58.3%) [30]. Previous studies have utilized bioterrorism course-based [31], computerized [32], and self-learning [33] education as an intervention and assesses its impact on the participants’ knowledge and attitudes. These studies have shown that education has a positive effect on participants’ knowledge and attitude. Therefore, providing healthcare professionals with the necessary knowledge and training would improve their skills and perform better in bioterrorism attacks.

Due to the rarity of the bioterrorism attacks, interest of health care workers in training, and gap in training as evidenced by our study findings, we suggest the institutional disaster management committees conduct annual emergency mocks/drills to promote readiness and sustain health care workers’ knowledge towards emergency disasters, including bioterrorism attacks. Based on the patterns of wrong answers of our study participants, revealed that health had information deficits regarding bioterrorism agents, routes of exposure, proper management of injuries and illnesses resulted from bioterrorism attacks, and prevention strategies.

The present study contributes to the previous literature in several ways. To the best of our knowledge, this is the first exploratory study in Saudi Arabia that investigates the levels of knowledge and preparedness of clinical laboratories and ED healthcare providers for bioterrorism-associated events. The use of knowledge-based multiple choice and true or false tests supported our study as well. It is recommended to develop educational programs in various formats, such as continuing education, undergraduate courses, online courses, and workshop training programs, to fulfill healthcare professionals’ needs.

The article is limited to the KSA situation but it could offer a more vital link to other policies on the matter or comparative analysis with other neighboring countries. A limitation of this study is that the cause-and-effect relationship among study variables could not be measured due to the cross-sectional design. Such a design can reflect respondents’ attitudes towards perceived benefits and barriers to getting bioterrorism preparedness education. Therefore, future research should focus on strategies that allow the prediction of the best education programs to prepare healthcare professionals better to respond to a bioterrorism attack.

## Conclusion

In conclusion, although healthcare professionals reported their willingness to assist in bioterrorism events, they need to enhance their knowledge of bioterrorism preparedness. The current study outlined various barriers of healthcare professionals to receiving bioterrorism preparedness education. Such obstacles may lead to unsatisfactory healthcare services for patients. Therefore, healthcare organizations must work together to reduce these barriers.

## Supplementary Information

**Additional file 1.**

## Data Availability

Data are available with the corresponding author.

## Abbreviations

COVD19: Coronaviruses disease 2019
CDC: Disease Control and Prevention
ED: Emergency department
EMD: Emergency medical services
FPC: Finite population correction
MCQ: Multiple-choice questions
AAMC: Association of American Medical Colleges
