# Supplementary material for: Knowledge and preparedness of healthcare providers towards bioterrorism
Source: BMC Health Serv Res. 2021 May 5;21:426. doi: 10.1186/s12913-021-06442-z (PMC8097244; doi:10.1186/s12913-021-06442-z)
Supplement: Supplementary file 1 — Additional file 1. [file 12913_2021_6442_MOESM1_ESM.docx]

**Title: Knowledge and Preparedness of Healthcare Providers towards Bioterrorism**

| **Part I:Demographic information:** |
| --- |
| - **Department/unit**   ☐Adult Emergency ☐ Pediatric Emergency ☐ Poison control center  ☐ EMS ☐Paramedic☐ Other, ………………….. |
| - **Profession:**   ☐Consultant ☐ Assistant consultant ☐ Resident ☐ Paramedic ☐ Head nurse/charge nurse ☐ Staff nurse ☐ Lab technique ☐ Other,……….. |
| - **Age: ……………years** |
| - **Sex:**   ☐ Male ☐ Female |
| - **Highest level of education:**   ☐ Diploma ☐ Bachelor degree ☐ Master’s degree ☐ Doctorate  ☐ Subspecialty or Fellowship |
| - **Total years of clinical experience at the current institution………………..years** - **Total years of clinical experience ………………..years** |
| - **Are you currently or have you ever been a member of your disaster planning committee?**   ☐ Yes ☐ No |
| - **Whom to contact during bioterrorism attack:**   ☐ Poison control center at the hospital ☐ 911 ☐ Other,………..   - **Are you aware about the hospital policy and procedure during bioterrorism attack?**   ☐ Yes ☐ No ☐ I don't know |

**Part II: Knowledge Questions**

| ***Please circle the best answer for the following questions***  **1. Which of the following diseases have potential for person-to-person spread?**  ☐ Anthrax and plague  ☐ Plague and botulism  ☐ Botulism and brucellosis  ☐ Smallpox and plague |
| --- |
| **2. Which of the following are good biological terrorism threats because of substantial morbidity and mortality, ease of production, efficient dissemination, stability in aerosol, or high infectivity?**  ☐ Anthrax, chickenpox, botulism, and plague  ☐ Anthrax, smallpox, chickenpox, and plague  ☐ Anthrax, smallpox, botulism, and plague  ☐ Anthrax, smallpox, mumps, and plague |
| **3. The deadliest form of anthrax is:**  ☐ Cutaneous  ☐ Inhalational  ☐ Gastrointestinal  ☐ Bubonic |
| **4. Which of the following symptoms is/are not commonly found in inhalation anthrax and if present could help to differentiate an upper respiratory tract infection from anthrax?**  ☐ Rhinorrhea and sore throat  ☐ Dyspnea  ☐ Meningeal signs  ☐ Vomiting  **5. A pathognomonic chest X-ray finding of advanced inhalation anthrax is:**  ☐ Cavitation  ☐ Widened mediastinum  ☐ Normal chest X-ray despite dyspnea and tachypnea |
| 6**. Smallpox has all of the following clinical features EXCEPT:**  ☐ The incubation period ranges from 7-17 days.  ☐ During the incubation period, the infected person looks and feels healthy and cannot infect others.  ☐ Infectivity is highest after the fever has begun and during the first 7-10 days following the appearance of the rash.  ☐ The virus can only be spread through direct or indirect contact with open lesions (e.g., by touching an infected lesion or by contact with infected clothing or bedding). |
| **7. Which of the following features help to distinguish the rash of smallpox from that of chickenpox:**  ☐ The initial smallpox lesions coincide with the onset of fever while the fever in chickenpox precedes the rash by 2-3 days.  ☐ The smallpox rash is centrifugal (majority of lesions on the face and extremities) while the rash in chickenpox is central (majority of lesions on the trunk).  ☐ Various stages of lesion progression can be found at any one single location on a smallpox patient while the lesions of chickenpox tend to be all at the same stage of development.  ☐ Lesions rarely occur on the palms and soles in smallpox, while lesions commonly occur on the palms and soles in chickenpox. |
| **8. What is a critical measure in preventing contact transmission of vaccinia virus (the agent used in the currently licensed smallpox vaccine)?**  ☐ Thorough hand washing after contact with the vaccination site  ☐ Isolation of the vaccinated person  ☐ Use of a porous bandage to cover the vaccination site  ☐ Antibacterial ointment applied to the vaccination site  ☐ Application of the vaccine at an anatomic site normally covered by clothing |
| **9. Epidemiologic features of a plague outbreak that may indicate an intentional release of the plague organism include:**  ☐Occurrence in persons with known health risks such as chronic pulmonary disease  ☐Occurrence in areas with prior reported rodent deaths  ☐Location of infections outside areas of known enzootic infection |
| **10. The most common early presenting syndrome associated with the majority of high risk (“Category A”) bioterrorism-associated diseases (i.e., anthrax, botulism, plague, smallpox, tularemia, and viral hemorrhagic fevers) is:**  ☐ Acute bloody diarrhea  ☐ Influenza-like illness  ☐ Acute hepatitis  ☐ Fever and rash |
| **11. Persistence of spores in the environment is of concern after a bioterrorism event involving:**  ☐ Anthrax  ☐ Tularemia  ☐ Plague  ☐ All of the above |
| **12. According to KFMC policies, a physician who sees a patient he or she suspects of having anthrax or smallpox must notify the Ministry of Health:**  ☐ By phone as soon as the suspected diagnosis has been laboratory confirmed  ☐ By phone as soon as the provisional diagnosis is established  ☐ By mail, phone, or fax within 72 hours  ☐ Immediately after receiving written permission from the patient (or his/her legal guardian) |

**Part II: Knowledge Questions**

**14. Please fill in or mark the box that best corresponds to your answer.**

|  | **True** | **False** |
| --- | --- | --- |
| 1. Procedures for biological and chemical patient decontamination are the same. |  |  |
| 1. Patient isolation should be based on the route of disease transmission. |  |  |
| 1. Guidelines about removing patients from isolation are the same after a bioterrorism attack as routine procedures. |  |  |
| 1. Personal protective equipment should be chosen based on the task being performed and the patient’s isolation precautions category. |  |  |
| 1. Environmental decontamination procedures depend upon the agent released. |  |  |
| 1. Only bleach should be used to disinfect environmental sources indoors following a bioterrorism attack. |  |  |
| 1. Young children and the elderly are two of the most vulnerable populations to the effects of a bioterrorism attack. |  |  |
| 1. The response actions for emerging infections, such as SARS and monkeypox, are very different from those for bioterrorism. |  |  |
| 1. A sudden influx of patients with flu-like symptoms may be the earliest indication of a bioterrorism attack. |  |  |
| 1. Bioterrorism attacks must not be reported until they are confirmed. |  |  |
| 1. Suspected bioterrorism attacks should be reported to the local health department. |  |  |
| 1. Your routine job duties will not be impacted by a bioterrorism attack. |  |  |
| 1. Weather conditions can affect the length of time that aerosolized biological particles remain airborne. |  |  |
| 1. Plans for back-up transportation should be arranged as part of nurses’ response plan. |  |  |
| 1. Chain of custody documentation is required for tracking patient specimens following a bioterrorism attack. |  |  |
| 1. Both acute and long-term mental health effects, such as anxiety and Post Traumatic Stress Disorder, can be expected to rise after a bioterrorism attack. |  |  |
| 1. Only police, emergency medical services, and fire protection professionals will use the incident command system to communicate during a bioterrorism attack. |  |  |
| 1. Biological agents can be dispersed via food, water, direct contact, or through aerosolization. |  |  |
| 1. The four phases of emergency management include: mitigation, preparedness, response, and recovery. |  |  |
| 1. Quarantine will be instituted after a bioterrorism attack involving any contagious disease. |  |  |
| 1. Duct-taping your windows will prevent the infiltration of infectious particles into your house following an aerosol release. |  |  |
| 1. All patients infected with a disease will have symptoms. |  |  |
| 1. Prompt initiation of post-exposure prophylaxis will prevent all patients from developing disease. |  |  |
| 1. A recent travel history, occupation, and vaccination history of victims will be needed as part of the epidemiological investigation of a bioterrorism attack. |  |  |
| 1. Use of alcohol-based products is an effective means of removing debris from the hands of victims exposed to a biological agent. |  |  |
| 1. Airborne spread diseases require the use of a negative pressure room in all settings. |  |  |
| 1. Staff caring for patients with diseases spread by respiratory droplets must wear N-95 masks. |  |  |
| 1. If you have been vaccinated against the disease that the patient has, you do not need to wear personal protective equipment when providing nursing care to them. |  |  |
| 1. Staff do not need a personal response plan for bioterrorism because their facility will have a disaster plan. |  |  |
| 1. Immunocompromised individuals will be more at risk for disease following a bioterrorism attack than young, healthy adults. |  |  |
| 1. Many of the potential bioterrorism agents cause upper respiratory symptoms. |  |  |
| 1. A large number of patients presenting with a rapidly fatal disease may indicate a bioterrorism attack has occurred. |  |  |
| 1. Vaccination administration following a bioterrorism attack will be similar to day-to-day immunizations. |  |  |
| 1. It is unsafe to cohort patients (putting patients with the same disease in the same room) during response to a bioterrorism attack. |  |  |
| 1. If you have children, back-up childcare should be arranged as part of your bioterrorism response plan. |  |  |
| 1. Run-off water from patient decontamination following a bioterrorism attack must be contained. |  |  |
| 1. Patient specimens should be hand carried to the laboratory during response to a bioterrorism attack; automated tube systems should not be used. |  |  |
| 1. Patient decontamination for bioterrorism includes the use of bleach as a disinfectant. |  |  |

**Part III: Perceived benefits of bioter­rorism preparedness education and barriers**

Please mark the single best answer that reflects your current knowledge/level of competency.

|  |  | Strongly Disagree | Disagree | Neutral | Agree | Strongly Agree |
| --- | --- | --- | --- | --- | --- | --- |
|  | Getting better prepared for bioterrorism will decrease my chances of getting sick/dying after a bioterrorism attack. |  |  |  |  |  |
|  | Getting better prepared for bioterrorism will decrease my family’s risk of getting sick/dying after a bioterrorism attack. |  |  |  |  |  |
|  | Getting better prepared for bioterrorism will decrease my patients’ risk of getting sick/dying after a bioterrorism attack. |  |  |  |  |  |
|  | Getting better prepared for bioterrorism will increase my chances of detecting an attack before surveillance would recognize it. |  |  |  |  |  |
|  | Getting better prepared for bioterrorism makes me feel more safe. |  |  |  |  |  |
|  | Bioterrorism preparedness advances my knowledge. |  |  |  |  |  |
|  | I have no interest in bioterrorism preparedness. |  |  |  |  |  |
|  | Bioterrorism preparedness is not currently a priority for me. |  |  |  |  |  |
|  | There are no training opportunities available on bioterrorism preparedness. |  |  |  |  |  |
|  | There are no bioterrorism-related disaster exercises available. |  |  |  |  |  |
|  | I do not know where to get bioterrorism preparedness training. |  |  |  |  |  |
|  | There is no administrative financial support for bioterrorism preparedness training for me at my work. |  |  |  |  |  |
|  | Bioterrorism training is all the same; I am not learning anything new. |  |  |  |  |  |
|  | Bioterrorism training is too expensive. |  |  |  |  |  |
|  | Bioterrorism training will take too long. |  |  |  |  |  |
|  | Bioterrorism preparedness is not within the scope of my responsibilities. |  |  |  |  |  |
|  | I feel uncomfortable/stressed when thinking about bioterrorism. |  |  |  |  |  |
|  | There is little one can do to lessen the impact of a bioterrorism attack. |  |  |  |  |  |
|  | My work schedule does not provide time for bioterrorism training. |  |  |  |  |  |
|  | I am too busy for bioterrorism training. |  |  |  |  |  |

| **Part IV: Training** |
| --- |
| - **I received prior training in bioterrorism preparedness:**   ☐ Yes ☐ No ☐ I'm not sure |
| - **In the event of a bioterrorist attack, I would be willing to provide assistance in the institution's response and control**   ☐ Yes ☐ No ☐ I'm not sure☐ No |

| **Part V: Which of the following areas do you feel you have a need for training? Please mark all that apply:** | |
| --- | --- |
| ☐ | 1. Recognition of an illness or injury in humans as potentially resulting from exposure to a bioterrorist agent |
| ☐ | 1. Surveillance (including syndromic surveillance) for bioterrorist agent |
| ☐ | 1. Laboratory diagnosis of bioterrorist agent |
| ☐ | 1. Safety measures to be taken by a public health responder in a bioterrorist event, including the use of protective equipment |
| ☐ | 1. Isolation and decontamination procedures |
| ☐ | 1. How to access clinical information about bioterrorist |
| ☐ | 1. Who to call if a bioterrorist event is suspected |
| ☐ | 1. How the public health system works in Saudi Arabia |
| ☐ | 1. Basic education regarding biological incidents |
| ☐ | 1. Disease investigation and reporting/epidemiologic methods |
| ☐ | 1. Hospital laws and statutes relating to public health measures |
